# A “giant bomb” in acute myocardial infarction due to coronary artery aneurysm: a case report

**DOI:** 10.3389/fcvm.2025.1631992

**Published:** 2025-07-14

**Authors:** Jin Chen, Wei Du, Jianyu Jiang, Liugang Xu

**Affiliations:** ^1^ Department of Cardiology, Zhangjiagang Hospital of Traditional Chinese Medicine Affiliated to Nanjing University of Traditional Chinese Medicine, Zhangjiagang, Jiangsu, China; ^2^ Department of Cardiology, Zhangjiagang City Hospital of Traditional Chinese Medicine Attached to Jiangsu Institute of Vocational Medicine, Zhangjiagang, Jiangsu, China

**Keywords:** coronary artery aneurysm, acute coronary syndromes, thrombosis, anticoagulation, case report

## Abstract

**Background:**

Coronary artery aneurysm associated with acute coronary syndromes represents a rare clinical entity, particularly in cases with persistent significant thrombus burden following aspiration therapy. The novel pathomechanism of intra-aneurysmal thrombosis secondary to pathological flow dynamics—characterized by endothelial shear stress perturbation and blood stasis—has been rarely documented. While existing literature describes therapeutic applications of surgical repair, percutaneous intervention, and pharmacotherapy in coronary artery aneurysm management, no consensus-driven protocol has been established, reflecting critical knowledge gaps in risk-stratified treatment algorithms for thrombus-laden aneurysms.

**Case outline:**

A 51-year-old female presenting with acute ST-segment elevation myocardial infarction underwent emergency coronary angiography, revealing a large right coronary artery aneurysm with significant intra-aneurysmal thrombus burden. Despite successful thrombus aspiration restoring distal TIMI 3 flow, persistent residual thrombus within the aneurysmal segment prompted the selection of intensified anticoagulation combined with dual antiplatelet therapy over percutaneous intervention. Throughout the therapeutic course, the patient remained asymptomatic without recurrent angina. Follow-up angiography performed after 7 days of targeted antithrombotic therapy demonstrated near-complete resolution of the intra-aneurysmal thrombus, accompanied by preserved coronary flow and no evidence of distal embolization.

**Conclusion:**

We present a rare case of ST-segment elevation myocardial infarction complicated by a coronary artery aneurysm, highlighting the therapeutic challenges in managing such complex pathologies. While intensified anticoagulation combined with dual antiplatelet therapy constitutes a clinically valid approach, the absence of consensus guidelines results in significant variability in treatment protocols. This case underscores the urgent need for large-scale multicenter studies to establish evidence-based management strategies for patients with coronary artery aneurysm associated with acute coronary syndromes.

## Introduction

Coronary artery aneurysm (CAA) is usually defined as localized coronary artery vasodilatation exceeding 1.5 times the diameter of the adjacent normal vessel (1), with an overall prevalence of 0.35%. They are most often detected during a coronary computed tomography angiography (CTA) or coronary angiography. Giant coronary artery aneurysms can have vessel diameters of 20, 40, 50 mm or more than four times the normal vessel diameter (2). Although the majority of patients with CAA are asymptomatic, previous studies have revealed that giant coronary artery aneurysm (GCAA) can lead to complications such as localized thrombosis, myocardial ischemia, rupture, and cardiac arrhythmias (3). Surgical, interventional, and pharmacological modalities have been applied to the treatment of these patients. Still, a uniform treatment protocol has not been developed (3, 4), especially in patients with CAA complicating acute coronary syndromes (ACS), where there is doubt as to whether direct percutaneous coronary intervention (PCI) should be performed.

This case report describes a 51-year-old female patient with ST-segment elevation myocardial infarction (STEMI) caused by a GCAA in the right coronary artery (RCA), identified during emergency coronary angiography. Following thrombus aspiration to restore blood flow (TIMI grade 3), significant residual thrombus was observed within the aneurysmal segment. Ultimately, intensified anticoagulation combined with dual antiplatelet therapy (DAPT) was selected over PCI. Follow-up coronary angiography confirmed satisfactory thrombus resolution and sustained coronary perfusion.

## Case presentation

**Figure 1:**
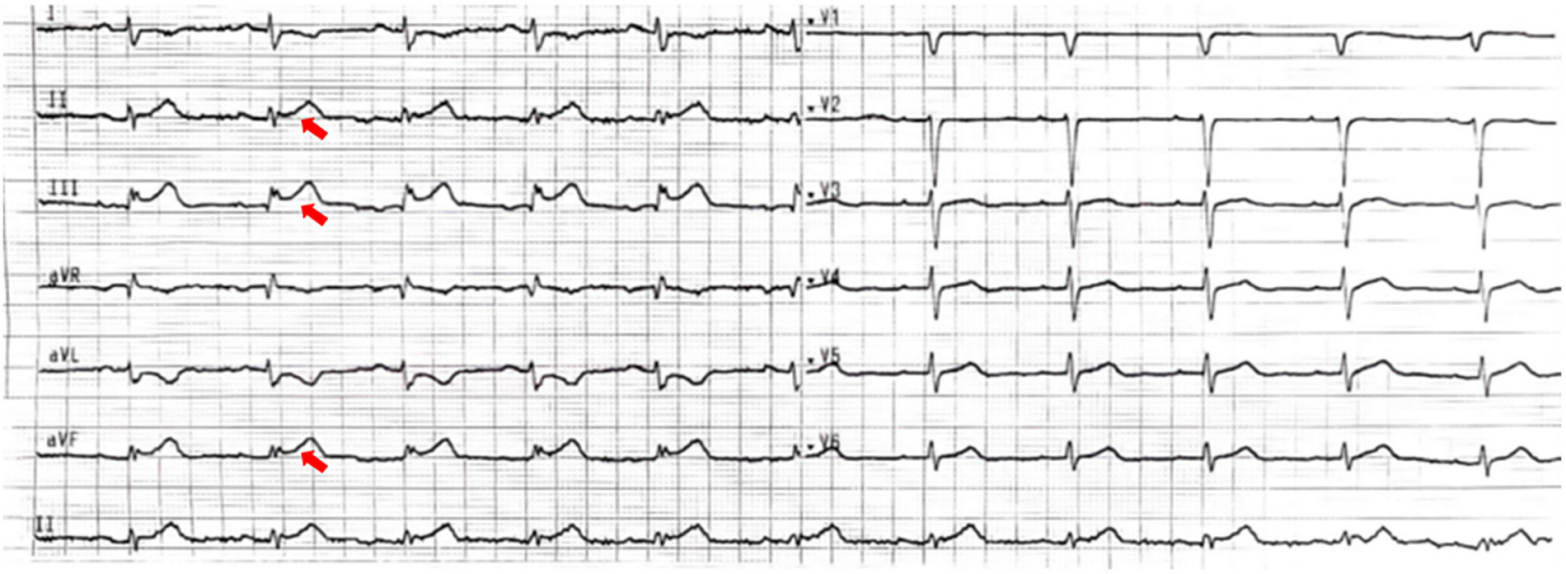
The electrocardiogram. The 12-lead electrocardiogram shows ST-segment elevation in leads II, III, and aVF (red narrow), with the ST-segment depression in the corresponding leads (I and aVL).

**Figure 2:**
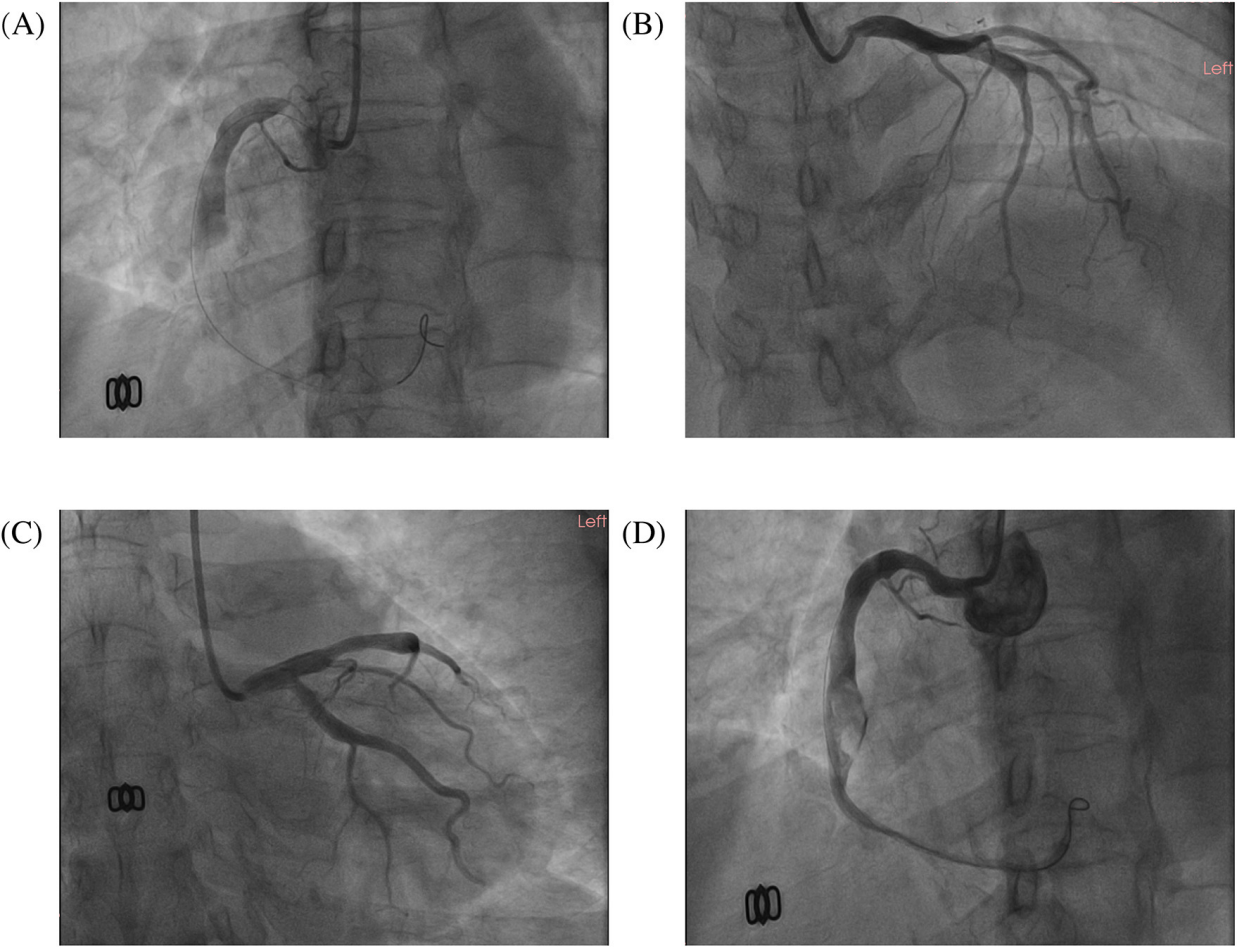
The images of the emergency coronary angiogram. The images of this patient's emergency coronary angiography. **(A)** LAO 45°; **(B)** RAO 30°+ CRA 20°; **(C)** RAO 30°+ CAU 20°; **(D)** the image after thrombus aspiration, LAO 45°.

**Figure 3:**
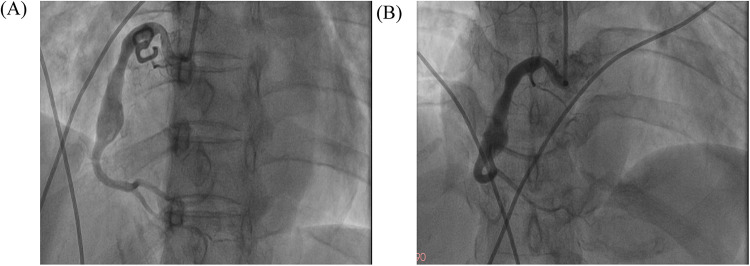
The images of follow-up coronary angiography. The images of follow-up coronary angiograms after the patient received intensive pharmacotherapy. **(A)** LAO 45°; **(B)** AP + CRA 20°.

A 51-year-old female presented to the emergency department with acute-onset chest pain persisting for 2 h. Medical history was negative for Kawasaki disease, with erythrocyte sedimentation rate and autoimmune markers (including antinuclear antibodies and anti-neutrophil cytoplasmic antibodies) within normal limits. The patient had no relevant previous history of coronary intervention. The electrocardiogram revealed ST-segment elevation in leads II, III, and aVF(red narrow), consistent with acute inferior wall myocardial infarction (Figure 1). Following immediate loading doses of aspirin 300 mg, ticagrelor 180 mg, and rosuvastatin 10 mg, and subsequent application of preoperative unfractionated heparin (UFH) (6,000 IU), the target anticoagulation dose was achieved based on the patient's weight. Emergent coronary angiography demonstrated: (1) Critical pathology was identified in the RCA, with a giant aneurysm (23 × 16 mm) in the mid-segment containing a massive thrombus burden, accompanied by TIMI 1 flow (Figure 2A); (2) The left anterior descending artery exhibited aneurysmal dilation in its proximal-to-mid segments while maintaining TIMI 3 flow (Figure 2B); (3) The left circumflex artery showed no significant stenosis with TIMI 3 flow (Figure 2C).

Sequential intracoronary administration of tirofiban (0.5 mg) and urokinase (100 kU) failed to resolve thrombotic occlusion. Subsequent thrombus aspiration retrieved substantial fresh thrombus, restoring TIMI 3 flow in the distal RCA. However, residual thrombus persisted within the aneurysmal segment (Figure 2D). The procedure was terminated upon resolution of chest pain and ST-segment normalization. The postoperative antithrombotic regimen comprised DAPT with aspirin 100 mg daily, ticagrelor 90 mg twice daily, and subcutaneous enoxaparin sodium 4,000 IU administered every 12 h.

Repeat coronary angiography performed one week of intensive antithrombotic therapy demonstrated near-complete dissolution of the intracoronary thrombus within the aneurysmal segment, accompanied by restored laminar flow in the affected right coronary artery (Figure 3). However, the patient declined to undergo intravascular ultrasound for precise assessment of the intracoronary aneurysm due to financial constraints. In light of the potential for recurrent thrombosis, we determined that stenting should be recommended following the patient's discharge from the hospital, with adequate anticoagulation treatment. Throughout the hospitalization, the patient remained free from anginal symptoms or ischemic electrocardiographic changes. Upon discharge, a tailored regimen combining therapeutic-dose rivaroxaban (10 mg daily) with DAPT (aspirin 100 mg daily and clopidogrel 75 mg daily) was initiated. Surveillance at 1- and 3-month post-discharge follow-ups revealed no adverse cardiovascular events, including recurrent thrombosis, myocardial infarction, or bleeding complications, with echocardiography confirming preserved left ventricular ejection fraction (LVEF 64%). Based on this favorable clinical course, the antithrombotic regimen was transitioned to rivaroxaban 10 mg daily plus clopidogrel 75 mg daily following the three-month evaluation.

## Discussion

CAA arises from diverse etiologies, with atherosclerosis, Kawasaki disease, polyarteritis nodosa, connective tissue disorders, and iatrogenic complications constituting the predominant causes in adults. While non-invasive modalities (e.g., echocardiography, CTA, and cardiac magnetic resonance) are clinically valuable (5, 6), invasive coronary angiography remains the diagnostic gold standard for definitive CAA evaluation. Large-scale registry data indicate a predilection for right coronary artery involvement (40%–70% of cases), followed by the left anterior descending (32.3%) and left circumflex arteries (7) (23.4%), while multivessel involvement is uncommon (<5% of cases). The patient denied any history of Kawasaki disease, with rheumatological and vasculitic markers showing no evidence supporting a diagnosis of autoimmune disorders. Coronary angiography revealed multivessel involvement with varying severity across all three major coronary arteries; however, the giant intra-aneurysmal thrombus within the RCA was identified as the causative factor for the STEMI.

CAA constitutes a significant risk factor for ACS (8, 9) through pathophysiological mechanisms involving endothelial discontinuity and flow stagnation within aneurysmal segments, which promote platelet activation and thrombogenesis. Data from the Coronary Artery Aneurysm Registry (1) reveal that 0.22% (*n* = 966/436,467) of ACS patients exhibited concurrent CAA during emergent angiography, with 69% undergoing PCI. Notably, 49.6% of these PCI-treated patients experienced major adverse cardiovascular events (MACE) within 37-month follow-up. Seong's report (10) indicates no-reflow phenomenon following PCI in a coronary aneurysm-associated STEMI case, endovascular interventions in thrombogenic aneurysmal segments carry substantial risks. In this case, persistent chest pain with unresolved ST-segment elevation indicated failure of coronary thrombus dissolution. These clinical manifestations suggested a predominantly thrombotic pathogenesis, prompting sequential intraprocedural administration of glycoprotein IIb/IIIa inhibitors and urokinase for targeted thrombolysis. Given the patient's preoperative anticoagulation with UFH 6,000 IU, we performed rigorous bleeding risk stratification before delivering a low-dose tirofiban bolus (10 μg/kg) combined with intracoronary urokinase (250 kU/20 ml). We attempted revascularization in this way while minimizing hemorrhagic complications, however, the effect is not immediately apparent. Eventually, a large amount of thrombus remained after thrombus aspiration on emergency coronary angiography, and anticoagulation combined with DAPT is likely to be more appropriate in this case, considering that implantation of a stent has the potential to cause acute in-stent thrombosis, in-stent restenosis, and no-reflow.

The treatment of CAA encompasses surgical intervention, percutaneous procedures, and pharmacotherapy (11), with therapeutic strategies tailored to individual patient characteristics. However, standardized protocols remain undefined due to limited consensus (12). CAA complicated by STEMI represents a rare clinical entity posing significant therapeutic challenges. Stent implantation in aneurysms with persistent thrombus substantially elevates the risks of acute in-stent thrombosis and no-reflow phenomenon. A case-control study by Schramet (13) identified CAA as an independent predictor of post-PCI no-reflow in STEMI patients. For high-risk cases with substantial thrombus burden and elevated no-reflow risk, deferred stenting following intensive antithrombotic therapy may constitute a viable approach. Emerging evidence supports optimized anticoagulation regimens for reducing cardiovascular events in CAA-associated ACS. Notably, a multicenter study involving 1,698 acute myocardial infarction patients demonstrated that adequate oral anticoagulation significantly lowered MACE incidence in those with concomitant coronary artery dilation (14).

The role of dual antiplatelets or therapeutic anticoagulation in the management of patients with CAA, particularly those with comorbid ACS, remains a subject of ongoing debate. However, the current state of evidence does not allow for either confirmation or refutation of the hypothesis that an escalated antiplatelet or antithrombotic regimen would be beneficial for these patients. Even if the existence of evidence suggests a potential benefit from anticoagulation in patients with CAA and ACS, robust clinical trials are warranted to determine the optimal anticoagulation strategy—warfarin vs. new oral anticoagulants (NOACs) such as rivaroxaban, apixaban, or edoxaban—for CAA-associated ACS management. A clinical trial (15) examining warfarin in patients with CAA showed that warfarin reduces the composite endpoint of UA, myocardial infarction, and aneurysm thrombosis in patients with CAA. In contrast, some cases (16, 17) have been reported in which the use of rivaroxaban (10–20 mg daily) has also been shown to achieve the goals of anticoagulation therapy. In our case, as this patient suffered from CAA combined with STEMI at the same time and was still given DAPT at the time of discharge, weighing the risk of bleeding as well as the efficacy of antithrombotic therapy, we finally applied rivaroxaban 10 mg daily.

In this case, a GCAA in the RCA induced pathological flow dynamics, promoting stasis and subsequent coagulation cascade activation (18). This hemodynamic disturbance culminated in localized thrombus formation and progression to STEMI. Given the persistent intra-aneurysmal thrombus burden observed post-thrombus aspiration, we prioritized intensified anticoagulation combined with DAPT over mechanical intervention. This conservative strategy aimed to mitigate the risks of stent thrombosis and no-reflow associated with endovascular prostheses in thrombogenic environments. Follow-up angiography performed after 7 days of targeted antithrombotic therapy demonstrated complete resolution of the aneurysmal thrombus, with restored TIMI 3 flow and no residual filling defects. The patient expressed high satisfaction with the efficacy of conservative pharmacotherapy and was ultimately discharged from hospital care. According to the current follow-up results, the antithrombotic regimen was transitioned to rivaroxaban 10 mg daily plus clopidogrel 75 mg daily following the three-month evaluation. To adequately assess the efficacy of this treatment protocol for such patients, it is imperative that long-term follow-up data be obtained and thoroughly analyzed.

## Conclusion

We present a rare case of STEMI complicated by a GCAA, highlighting the therapeutic challenges in managing such complex pathologies. While intensified anticoagulation combined with DAPT constitutes a clinically valid approach, the absence of consensus guidelines results in significant variability in treatment protocols. This case underscores the urgent need for large-scale multicenter studies to establish evidence-based management strategies for patients with CAA associated with ACS.

## Data Availability

The original contributions presented in the study are included in the article/Supplementary Material, further inquiries can be directed to the corresponding author.
